# Declining Accuracy in Disease Classification on Health Insurance Claims: Should We Reconsider Classification by Principal Diagnosis?

**DOI:** 10.2188/jea.JE20090044

**Published:** 2010-03-05

**Authors:** Etsuji Okamoto

**Affiliations:** National Institute of Public Health, Department of Management Sciences, Wako, Saitama, Japan

**Keywords:** health insurance claims, ICD10, intracategory variance, intercategory variance, log-normal distribution

## Abstract

**Background:**

An ideal classification should have maximum intercategory variance and minimal intracategory variance. Health insurance claims typically include multiple diagnoses and are classified into different disease categories by choosing principal diagnoses. The accuracy of classification based on principal diagnoses was evaluated by comparing intercategory and intracategory variance of per-claim costs and the trend in accuracy was reviewed.

**Methods:**

Means and standard deviations of log-transformed per-claim costs were estimated from outpatient claims data from the National Health Insurance Medical Benefit Surveys of 1995 to 2007, a period during which only the ICD10 classification was applied. Intercategory and intracategory variances were calculated for each of 38 mutually exclusive disease categories and the percentage of intercategory variance to overall variance was calculated to assess the trend in accuracy of classification.

**Results:**

A declining trend in the percentage of intercategory variance was observed: from 19.5% in 1995 to 10% in 2007. This suggests that there was a decline in the accuracy of disease classification in discriminating per-claim costs for different disease categories. The declining trend temporarily reversed in 2002, when hospitals and clinics were directed to assign the principal diagnosis. However, this reversal was only temporary and the declining trend appears to be consistent.

**Conclusions:**

Classification of health insurance claims based on principal diagnoses is becoming progressively less accurate in discriminating per-claim costs. Researchers who estimate disease-specific health care costs using health insurance claims must therefore proceed with caution.

## INTRODUCTION

Health insurance claims contain diagnostic information and are a valuable data source for economic and epidemiological studies. However, 2 problems arise when researchers use health insurance claims for epidemiological studies: the need to ensure (1) the accuracy of diagnoses and (2) the accuracy of disease classification. The former is challenging because health insurance claims are essentially financial documents and not medical records. The latter derives from the fact that principal diagnoses are chosen rather arbitrarily when the coders are not properly trained.

To bypass these difficulties, studies attempting to evaluate the economic effects of smoking,^1^ walking,^2^ and health promotional activities^3^ have largely used per-capita health care cost, without disease classification. Some studies estimating disease-specific health care costs for diseases such as asthma^4^ and liver disease^5^ also used other data sources, including the Patient Survey (a one-day cross-sectional sampling survey conducted by the Japanese Ministry of Health, Labour & Welfare), to increase the accuracy of disease classification. Indeed, disease classification on health insurance claims was shown to be of questionable accuracy when compared with the Patient Survey even for a well-defined disease category like dialysis.^6^

Health insurance claims are widely used for epidemiological studies abroad, and foreign researchers have validated the accuracy of diagnoses in an empirical manner with more or less positive results. A Korean study reported 76% accuracy of acute myocardial infarction (AMI) diagnoses through matching with medical records.^7^ A Taiwan study reported 74.6% accuracy of diabetes diagnoses through a questionnaire survey to patients.^8^ Researchers in the United States reported even higher accuracy: 94.1% positive predictive value (PPV) for AMI diagnoses,^9^ 72.6% to 80.8% PPV for pneumonia,^10^ and 76.2% sensitivity and 93.3% specificity for hypertension.^11^ Some researchers went so far as to match cases with the cancer registry to validate diagnoses of malignancy.^12^

However, when researchers use health insurance claims classified by principal diagnoses, the second problem, ie, the accuracy of classification, is more important than the accuracy of diagnoses per se. There may also be systematic biases in classification, because some diseases are more likely to be chosen as principal diagnoses than others.^13^

Accuracy of diagnoses can only be validated empirically through matching with a gold standard such as medical records, but accuracy of disease classification can be evaluated statistically. If claims of the same disease category have the same values, accurate classification should yield uniform claims, ie, zero variance. In other words, accurate classification should maximize the intercategory variance while minimizing intracategory variance.

In this study, statistical analysis is used to evaluate the accuracy of classification by analyzing per-claim costs of outpatient claims. Per-claim cost is the amount of money charged for medical treatment and is written on the bottom line of a health insurance claim. Per-claim cost is expressed in points and can be converted into Japanese yen by multiplying by 10.

## METHODS

### Theory

Disease-specific means and variance can be estimated from published frequency tables—without referring to microdata that are not readily available—by using an optimization program such as Excel Solver with the assumption of a particular distribution. If a normal distribution is assumed, as is usually the case, then the frequency tables must follow a normal distribution for the optimization program to yield good estimates. Per-claim costs of health insurance claims do not follow a normal distribution, as evidenced by the skewed distribution in the frequency tables; they follow a log-normal distribution.^14^ Therefore, the ranges of frequency tables were log-transformed to ensure normal distribution. The goodness-of-fit of the log-normal distribution was confirmed by the Kolmogorov-Smirnov test.

### Data source

The data source was the National Health Insurance Medical Benefit Survey (NHIMBS), a sampling survey on health insurance claims submitted in May of the survey year. Japan’s National Health Insurance covers the population that does not have regular employment, eg, the self-employed, retired, and part-time workers. The NHIBMS has been administered by the Ministry of Health, Labour & Welfare (MHLW), Bureau of Health Insurance, Department of Investigation every year since 1955. The reports include 24 summary tables and 11 raw output tables, which are distributed by the Central Federation of National Health Insurance. Summary tables from 1998 to 2005 and both summary and raw output tables from 2005 to 2007 are available from the Portal Site of Official Statistics of Japan, maintained by the Statistics Bureau, Ministry of Internal Affairs and Communications, with the collaboration of related ministries and agencies (http://www.e-stat.go.jp).

The NHIMBS is a survey of all insurers (1818 municipal governments and 165 National Health Insurance societies as of March 2007). Health insurance claims are sampled randomly by each insurer at a specified sampling proportion. The sampling proportion is approximately 1/500 for regular and elderly beneficiaries. Until 2002, elderly was defined as age 70 years or older, after which the threshold was raised gradually to 75 years in 2007. Elderly beneficiaries also include people 65 years or older with certain disabilities. The sampling proportion for retiree beneficiaries is 1/100.

Because health insurance claims are administrative data, the population of health insurance claims can be determined from monthly administrative reports compiled by the Central Federation of National Health Insurance (www.kokuho.or.jp). The exact population and sample size of outpatient claims, as well as the number of beneficiaries from which the data were derived, are shown in Table 1. Thirteen years of data (1995–2007) were used because the same ICD10 classification (commonly referred to as the “119 classification”^15^) has been applied since 1995.

**Table 1.** Data on beneficiaries and outpatient claims

	No. of beneficiaries^a^				No. of outpatient claims					
		
	(in thousands)				Regular and elderly beneficiaries			Retiree beneficiaries		
						
	Regular	Retiree	Elderly	Total	Population (P)^a^	(Sample (S)^b^)	P/S	Population (P)^a^	(Sample (S)^b^)	P/S
1995	30 507	4153	8290	42 950	23 315 377	(47 572)	490.1	3 660 465	(36 707)	99.7
1996	30 319	4254	8831	43 404	24 472 909	(49 297)	496.4	3 840 657	(36 303)	105.8
1997	30 451	4373	9352	44 176	25 123 382	(48 647)	516.4	3 935 421	(39 472)	99.7
1998	30 155	4590	9921	44 666	26 074 183	(52 711)	494.7	4 144 267	(41 694)	99.4
1999	30 520	4786	10 542	45 848	27 134 887	(55 406)	489.7	4 315 181	(43 426)	99.4
2000	30 710	5140	11 190	47 040	29 160 510	(59 429)	490.7	4 741 168	(47 450)	99.9
2001	31 213	5343	12 396	48 952	30 485 879	(62 331)	489.1	4 949 316	(49 243)	100.5
2002	31 460	5531	12 567	49 558	31 434 551	(63 893)	492.0	5 028 551	(50 398)	99.8
2003	32 264	6047	12 591	50 902	32 250 061	(65 420)	493.0	5 463 517	(54 600)	100.1
2004	32 691	6795	12 204	51 690	31 511 976	(64 424)	489.1	6 035 495	(60 264)	100.2
2005	32 680	7500	11 730	51 910	32 192 705	(65 289)	493.1	6 949 882	(69 212)	100.4
2006	32 340	8190	11 270	51 800	32 516 241	(65 900)	493.4	7 878 017	(78 171)	100.8
2007	31 783	8822	10 819	51 424	32 368 390	(65 436)	494.7	8 727 709	(86 610)	100.8

^a^from monthly administrative reports compiled by the Central Federation of National Health Insurance.

^b^from National Health Insurance Medical Benefit Survey.

The representativeness of the data is believed to be satisfactory because the survey includes all insurers. However, some irregularities were observed for renal failures in 2000, as shown in Table 2. Data in the genitourinary disease category of 2000 were modified according to the 1999 and 2001 data.

**Table 2.** Distribution of claims of renal failure (ICD10:N00–19) by per-claim cost (%)

	Sample size (sampling proportion)			Per-claim cost (yen)							Arithmetic mean
		
	Regular beneficiaries (1/500)	Elderly beneficiaries (1/500)	Retiree beneficiaries (1/100)	1–500	500–1000	1000–2000	2000–3000	3000–5000	5000–10 000	>10 000	
1995	211	138	245	12.3	11.1	16.3	11.8	7.5	5.5	34.9	159 292
1996	211	165	211	14.1	9.1	17.0	10.5	7.7	5.3	35.6	163 785
1997	193	162	234	10.5	12.0	15.5	8.5	9.2	5.0	39.2	170 907
1998	217	185	267	10.5	9.5	19.8	9.2	7.9	5.7	36.7	168 703
1999	193	212	266	11.4	11.6	12.2	8.1	6.6	3.5	46.1	186 753
2000	219	247	287	13.8	10.3	17.8	8.6	7.5	***27.0***^a^	***14.5***^a^	***94 008***^a^
2001	219	240	314	12.2	10.7	16.5	8.0	6.4	5.2	41.0	177 358
2002	198	255	270	13.7	9.4	14.6	5.7	6.6	3.4	46.5	189 810
2003	194	269	314	15.7	10.0	14.6	6.4	5.6	5.2	42.4	174 586
2004	263	229	362	11.1	11.1	14.1	7.3	6.7	4.0	45.7	174 838
2005	248	289	377	14.6	12.7	13.4	6.1	5.6	4.9	42.6	166 683
2006	241	251	399	14.4	13.6	14.7	5.2	4.1	4.1	43.8	173 836
2007	227	260	428	11.2	12.9	12.3	6.6	6.8	3.3	47.0	184 728

^a^irregularities are shown in ***bold, italic font***.

source: National Health Insurance Medical Benefit Survey.

### Estimation of means and standard deviations of log-transformed per-claim costs

To determine whether an observed distribution follows a certain distribution (such as a normal distribution), the Kolmogorov-Smirnov (KS) test is used. In this test, the maximum discrepancy between the 2 cumulative distributions, or KS value, is used as a test statistic. If the KS value is smaller than 1.63/√n, then one can assume that the observed distribution follows the certain distribution at *P* = 0.01.^16^

Because the NHIMBS provides only arithmetic means for per-claim costs, the means (m) and standard deviations (σ) of log-transformed per-claim costs were estimated from disease-specific frequency tables (Summary table 16-2) using Excel Solver, an add-in program for Microsoft Excel software.

For example, 25.1% of outpatient claims with a principal diagnosis of diabetes were in the range of 500 to 1000 yen per-claim in 2006. The range of 500 to 1000 yen was log-transformed to LN(500)–LN(1000) or 6.21 to 6.91 (LN, natural logarithm). If the log-transformed per-claim costs follow a normal distribution, the proportion of claims in this range is expressed with Excel functions as follows (“TRUE” in Excel functions denotes cumulative density functions; “FALSE” denotes probability density functions):

+NORMDIST(6.91,m,σ,TRUE)−NORMDIST(6.21,m,σ,TRUE) [1]

Frequency tables consist of 7 ranges (1–500, 500–1000, 1000–2000, 2000–3000, 3000–5000, 5000–10 000, and ≥10 000 yen per-claim). Let R*_k_* denote the cumulative proportion of claims in the frequency tables in the *k*th range (1 ≦ *k* ≦ 7) and E*_k_* denote the estimated cumulative proportion in the log-transformed *k*th range using formula [1]. Then, the KS value is expressed as follows:

$$\mathrm{KS value}=\mathrm{MAX}|R_{k}-E_{k}|$$ [2]

Optimal m and σ were obtained using Excel Solver to minimize the KS value of the formula [2] for all disease categories and years. The square of σ, σ^2^, gives the variance within a given disease category (hereafter referred to as intracategory variance).

### Estimation of intercategory variances

Let n, m, and σ denote the number of claims, and the mean and standard deviation of per-claim costs, respectively, of an entire sample, and n*_k_*, m*_k_*, and σ*_k_* denote those of the *k*th disease category. The relationship between the entire sample and disease categories are expressed as follows:

$$n*m=\sum_{k}n_{k}*m_{k}$$ [3]

$$n*\sigma^{2}=\sum_{k}n_{k}*\sigma_{k}{}^{2}+\sum_{k}n_{k}*{\left(m_{k}-m\right)}^{2}$$ [4]

Formula [4] signifies the following relationship:

all variance=intracategory variance+intercategory variance [5]

Hence, intercategory variance was calculated using the second part of the right side of formula [4].

### Extrapolation of sample size

The number of sampled claims was obtained from the raw output tables (Table 7-1 for regular, 7-2 for elderly, and 7-3 for retiree beneficiaries) of the NHIMBS. However, the number of claims from these 3 beneficiary categories cannot be summed because the sampling proportion is different (1/500 for regular and elderly beneficiaries and 1/100 for retiree beneficiaries). Hence, the number of claims for retiree beneficiaries was deflated by five to adjust for the difference in sampling proportion.

### Calculation of means and variance of residual subcategories

The NHIMBS presents disease-specific data on all 19 major disease categories in ICD10 (I–XIX), plus some selected subcategories. For example, NHIMBS presents data on ophthalmic disease (VII), as well as on a subcategory—cataract (H25–26). From these data, a residual subcategory, “other ophthalmic diseases (H00–59 minus H25–26)”, must be extrapolated to create a mutually exclusive disease classification. The means and variances of residual subcategories can be calculated using formula [4]. A total of 38 mutually exclusive disease categories were thus created. A subcategory, “renal failure”, was merged with a major category, “genitourinary diseases”, because of irregularities in the data.

## RESULTS

Table 3 illustrates how the optimal m and σ were obtained. The left frequency table presents an actual distribution of per-claim costs and the right frequency table presents a theoretical distribution when per-claim costs are log-transformed and assumed to follow a normal distribution with optimal m and σ minimizing the KS value.

**Table 3.** Optimal means and standard deviations of log-transformed per-claim costs of outpatient claims

Year	Per-claim cost from NHIMBS							LN(per-claim cost) optimized to minimize KS values									
	
	Frequency table (%)							Frequency table (%)							KS value	Optimal	
		
	1–500	500–1000	1000–2000	2000–3000	3000–5000	5000–10 000	>10 000	0–6.21	6.21–6.91	6.91–7.60	7.60–8.01	8.01–8.52	8.52–9.21	9.21		mean	SD
1995	24.0	25.8	25.8	11.3	8.3	3.7	1.2	24.0	26.0	26.0	10.9	8.1	4.1	1.0	0.30	6.91	0.98
1996	22.5	26.0	26.4	12.0	8.3	3.6	1.2	22.2	26.2	26.9	11.3	8.4	4.1	0.9	0.34	6.95	0.96
1997	22.2	25.5	27.0	12.2	8.2	3.5	1.3	22.1	26.1	26.9	11.3	8.4	4.2	0.9	0.47	6.95	0.96
1998	24.3	26.5	27.8	10.6	6.6	2.9	1.3	24.0	27.3	26.6	10.6	7.4	3.4	0.7	0.68	6.88	0.94
1999	25.5	26.8	27.4	9.8	6.3	2.8	1.3	25.6	27.5	26.0	10.1	7.0	3.2	0.6	0.70	6.84	0.95
2000	25.3	26.3	27.8	10.3	6.6	3.1	0.7	24.7	27.6	26.5	10.3	7.1	3.2	0.6	0.64	6.85	0.94
2001	26.2	26.3	27.0	10.2	6.5	2.8	1.1	25.6	27.4	26.0	10.1	7.1	3.2	0.6	0.56	6.84	0.95
2002	28.0	26.5	27.1	9.3	5.6	2.4	1.0	27.2	28.1	25.5	9.6	6.4	2.7	0.5	0.78	6.78	0.94
2003	29.5	27.3	25.6	8.9	5.3	2.3	1.0	29.7	27.7	24.4	9.0	6.0	2.6	0.5	0.61	6.73	0.96
2004	30.9	27.3	24.8	8.6	5.0	2.2	1.1	30.8	27.6	23.9	8.8	5.9	2.6	0.5	0.71	6.70	0.97
2005	31.1	26.9	24.6	8.8	5.2	2.3	1.1	31.3	27.2	23.5	8.7	5.9	2.7	0.5	0.56	6.69	0.99
2006	32.0	26.8	24.4	8.5	5.0	2.2	1.1	31.8	27.4	23.4	8.6	5.8	2.6	0.5	0.60	6.68	0.98
2007	32.3	26.8	23.8	8.6	5.0	2.3	1.2	31.8	27.1	23.3	8.6	5.9	2.7	0.5	0.66	6.68	0.99

NHIMBS: National Health Insurance Medical Benefit Survey.

KS value: Kolmogorov-Smirnov value; SD: standard deviation.

Table 4 shows the results of the KS test for goodness-of-fit. Overall, per-claim costs were shown to follow a log-normal distribution in 5 of 13 years (1995, 1996, 1997, 2001, and 2005). On a disease-specific level, a majority of disease categories were shown to follow log-normal distributions. Most notably, all disease categories followed a normal distribution in 1995 and 1996. Hypertension had the largest number of non-compatible years (11 out of 13 years), reflecting its large sample size, followed by genitourinary diseases (9 out of 13 years), including dialysis, which has an exceptionally high per-claim cost. The overall compatibility improved when hypertension and/or genitourinary diseases were excluded (shown as a reference in Table 4). Without these 2 categories, per-claim costs were shown to follow a log-normal distribution in all 13 years.

**Table 4.** Results of Kolmogorov-Smirnov (KS) test for goodness-of-fit (α = 0.01)

Diagnostic categories	ICD 10	1995	1996	1997	1998	1999	2000	2001	2002	2003	2004	2005	2006	2007	No. of *
ALL	ALL				*	*	*		*	*	*		*	*	8
ALL except hypertension^b^											*		*	*	3
ALL except genitourinary diseases^b^							*	*	*	*					4
ALL except hypertension and genitourinary diseases^b^															0
gastrointestinal infection	A00–09														0
tuberculosis	A15–19														0
sexually transmitted disease	A50–64														0
other infectious diseases	rest of A00–B99														0
malignant tumor	C00–97												*	*	2
nonmalignant tumor	D00–48														0
hematopoietic diseases	D50–89														0
diabetes	E10–14														0
other endocrine diseases	rest of E00–90						*		*						2
schizophrenia	F20–29														0
other psychiatric diseases	rest of F00–99														0
neurological diseases	G00–99												*	*	2
cataract	H25–26												*	*	2
other ophthalmic diseases	rest of H00–59														0
diseases of the ear	H60–95														0
hypertension	I10–15			*	*	*	*	*	*	*	*	*	*	*	11
ischemic heart diseases	I20–25												*	*	2
cerebrovascular diseases	I60–69												*	*	2
other circulatory diseases	rest of I00–99														0
acute upper respiratory infection	J00–06														0
pneumonia	J12–18														0
acute bronchitis	J20–21												*	*	2
chronic sinusitis	J32														0
chronic obstructive pulmonary diseases	J40–44												*	*	2
asthma	J45–46														0
other respiratory diseases	rest of J00–99														0
stomach and duodenal diseases	K25–29														0
liver diseases	K70–77														0
other gastrointestinal diseases	rest of K00–93														0
skin diseases	L00–99														0
musculoskeletal diseases	M00–99														0
genitourinary diseases	N00–99				*	*		*	*	*	*	*	*	*	9
pregnancy and delivery	O00–99														0
perinatal conditions	P00–96														0
congenital anomalies	Q00–99														0
unspecified conditions	R00–99														0
bone fractures	^a^														0
other injuries	rest of S00–T98														0

No. of *		0	0	1	2	2	2	2	3	2	2	2	9	9	

*: significantly different from normal distribution at confidence level 0.01.

^a^S02,12,22,32,42,52,62,72,82,92,T02,08,10,12.

^b^for reference.

Table 5 and 6 show the exponentiated m and σ (exp(m) and exp(σ)) or geometric means and standard ratio for all disease categories and years. Geometric means of per-claim costs have consistently decreased, which may reflect a reduction in drug costs due to increasing separation of dispensing and prescription. In contrast, the standard ratio remains constant, around 2.55 to 2.70, throughout the study period. It is noteworthy that the standard ratio of per-claim costs is close to the Napier constant (*e* = 2.718). If the geometric mean of per-claim costs is 1000 yen and the standard ratio is 2.7, one can assume that 68% of claims fall within the range of 1000/2.7 to 1000 * 2.7, or 370 to 2700 yen.

**Table 5.** Exponentiated optimal means of log-transformed per-claim costs (= geometric means; in Japanese yen)

	ICD10	1995	1996	1997	1998	1999	2000	2001	2002	2003	2004	2005	2006	2007
ALL	ALL	**1002.3**	**1041.4**	**1044.9**	**969.8**	**930.7**	**948.4**	**930.8**	**882.9**	**835.6**	**813.6**	**807.9**	**795.7**	**798.8**
gastrointestinal infection	A00–09	681.4	689.8	715.5	668.7	620.5	662.7	665.6	622.8	695.9	631.6	630.2	577.1	614.4
tuberculosis	A15–19	1465.7	1798.0	1397.2	1465.6	1358.4	1403.6	1220.1	1295.5	1235.8	833.8	1306.1	1103.5	1248.9
sexually transmitted disease	A50–64	859.6	1086.5	914.6	748.1	866.7	1242.9	1037.7	1043.9	866.1	806.2	898.4	802.6	769.3
other infectious diseases	rest of A00–B99	739.7	756.3	844.2	820.5	823.5	756.3	763.7	735.9	696.3	674.7	710.0	709.9	699.5
malignant tumor	C00–97	2011.7	1917.1	1871.5	1620.5	1615.8	1454.0	1458.8	1462.0	1481.8	1431.5	1497.1	1382.0	1538.5
nonmalignant tumor	D00–48	1271.5	1313.7	1287.6	1242.0	1100.6	1091.9	1083.2	996.5	1205.6	1008.0	992.4	921.4	952.3
hematopoietic diseases	D50–89	998.7	936.0	1069.5	959.7	815.6	968.4	870.4	876.3	837.1	743.8	872.5	808.3	865.7
diabetes	E10–14	1594.9	1583.5	1604.8	1448.8	1388.4	1424.3	1406.5	1372.5	1285.7	1264.8	1237.0	1285.1	1276.0
other endocrine diseases	rest of E00–90	1260.1	1406.5	1301.9	1177.8	1107.4	1104.8	1106.7	1072.2	1031.6	958.7	947.7	921.4	914.5
schizophrenia	F20–29	1338.1	1307.8	1237.5	1248.4	1205.9	1151.4	1256.1	1186.7	1160.6	1044.6	1033.1	1022.7	948.0
other psychiatric diseases	rest of F00–99	1000.3	1063.3	1033.8	998.7	925.6	953.4	892.0	916.5	891.4	828.2	795.7	737.1	700.5
neurological diseases	G00–99	1017.4	1036.7	1077.2	945.5	872.7	923.5	925.2	756.4	779.9	752.3	777.3	759.4	733.4
cataract	H25–26	535.5	530.5	544.6	519.2	524.3	537.8	533.4	495.8	500.6	469.0	477.7	517.7	520.2
other ophthalmic diseases	rest of H00–59	574.7	602.4	588.5	594.1	594.8	586.7	585.1	555.7	568.3	551.5	563.0	537.8	528.9
diseases of the ear	H60–95	729.1	787.8	802.7	720.5	731.2	729.3	706.4	678.5	643.3	638.8	621.3	630.3	594.0
hypertension	I10–15	1308.8	1333.7	1359.9	1210.2	1184.2	1240.0	1236.8	1169.6	1065.2	989.7	986.8	975.4	947.0
ischemic heart diseases	I20–25	1517.1	1591.5	1556.4	1418.1	1327.3	1347.6	1274.3	1172.7	1077.9	1011.6	1024.4	1030.7	986.7
cerebrovascular diseases	I60–69	1711.2	1751.3	1796.6	1499.3	1334.6	1281.4	1166.9	1102.0	1009.7	936.3	950.4	961.7	902.3
other circulatory diseases	rest of I00–99	1296.5	1336.1	1289.6	1203.0	1165.3	1173.3	1156.1	1000.4	939.9	904.0	906.8	875.6	905.0
acute upper respiratory infection	J00–06	550.1	586.0	600.4	598.0	625.7	605.9	598.4	577.7	565.1	589.0	563.1	559.5	556.0
pneumonia	J12–18	1506.7	1941.8	1386.2	1243.8	1537.7	1488.0	1213.8	1363.6	1161.1	1194.6	1301.2	1295.7	1407.8
acute bronchitis	J20–21	691.0	740.3	771.2	714.9	720.4	708.9	703.7	633.0	660.2	669.0	645.8	608.9	614.6
chronic sinusitis	J32	729.9	774.3	795.0	765.2	741.0	741.9	685.5	700.7	637.4	601.4	658.1	587.9	603.6
chronic obstructive pulmonary diseases	J40–44	859.2	965.3	1051.4	984.8	921.0	917.1	907.8	951.7	829.1	877.7	751.8	756.6	758.1
asthma	J45–46	1105.8	1124.6	1104.3	1001.5	1020.9	969.2	975.3	853.1	830.6	865.4	810.9	761.4	778.7
other respiratory diseases	rest of J00–99	660.9	735.6	704.0	668.5	626.7	632.9	614.2	605.8	563.2	600.0	533.5	605.8	596.5
stomach and duodenal diseases	K25–29	1281.0	1263.6	1244.4	1176.1	1105.5	1127.7	1104.5	1052.7	969.6	937.6	955.2	942.9	915.9
liver diseases	K70–77	1573.4	1618.0	1526.6	1417.7	1398.7	1400.2	1384.9	1375.3	1425.5	1282.8	1240.5	1272.4	1252.9
other gastrointestinal diseases	rest of K00–93	956.5	999.9	1010.9	898.7	857.9	882.7	895.3	827.0	723.5	689.4	697.9	718.0	704.2
skin diseases	L00–99	488.3	506.8	506.5	513.2	489.8	486.1	474.5	431.2	452.0	411.7	422.7	425.1	391.1
musculoskeletal diseases	M00–99	1195.7	1184.8	1203.1	1105.4	1059.4	1077.1	1044.4	937.9	934.6	899.8	915.8	883.3	885.4
genitourinary diseases	N00–99	1180.3	1217.2	1212.8	1121.6	1071.2	1032.3	1001.4	947.9	884.4	833.8	845.4	859.0	832.2
pregnancy and delivery	O00–99	859.0	963.1	980.9	923.5	927.2	992.5	850.1	793.2	861.5	831.3	958.3	857.2	947.4
perinatal conditions	P00–96	728.0	585.9	578.5	564.8	887.0	689.5	636.7	615.1	687.1	525.5	767.3	955.7	931.1
congenital anomalies	Q00–99	744.0	808.0	856.0	695.4	823.3	676.6	693.1	831.0	578.9	701.4	650.9	632.3	594.4
unspecified conditions	R00–99	780.8	715.0	731.0	746.6	723.9	745.5	733.8	635.4	631.1	641.2	652.1	698.1	703.3
bone fractures	^a^	1219.3	1272.7	1250.0	1142.5	1247.7	1218.3	1115.7	1096.1	1096.3	979.9	1111.1	1108.7	1070.6
other injuries	rest of S00–T98	795.7	855.4	884.0	865.3	822.4	875.0	839.8	804.1	784.1	768.8	803.3	778.4	807.4

^a^S02,12,22,32,42,52,62,72,82,92,T02,08,10,12.

**Table 6.** Exponentiated optimal standard deviations of log-transformed per-claim costs (= standard ratio)

	ICD10	1995	1996	1997	1998	1999	2000	2001	2002	2003	2004	2005	2006	2007
ALL	ALL	**2.67**	**2.60**	**2.61**	**2.56**	**2.58**	**2.55**	**2.58**	**2.56**	**2.62**	**2.64**	**2.68**	**2.67**	**2.70**
gastrointestinal infection	A00–09	2.71	2.28	2.39	2.47	2.29	2.29	2.29	2.17	2.32	2.06	2.41	2.18	2.33
tuberculosis	A15–19	2.46	2.59	2.66	2.58	2.86	2.71	2.74	3.39	3.62	3.44	2.81	3.07	3.59
sexually transmitted disease	A50–64	2.31	2.99	2.57	2.28	2.92	2.22	1.94	1.95	1.98	2.13	2.00	1.94	2.33
other infectious diseases	rest of A00–B99	3.06	2.98	2.82	2.84	2.71	2.81	2.73	2.80	2.79	2.92	2.77	2.70	2.79
malignant tumor	C00–97	3.34	3.39	3.19	3.68	3.61	3.25	3.51	3.91	4.02	4.12	3.80	3.94	4.02
nonmalignant tumor	D00–48	3.03	3.21	3.22	3.15	3.35	3.06	3.20	3.22	3.06	3.24	3.18	2.92	3.38
hematopoietic diseases	D50–89	2.47	3.05	2.87	2.61	2.77	2.75	2.63	2.64	2.38	2.93	2.74	2.53	2.66
diabetes	E10–14	2.38	2.29	2.32	2.30	2.38	2.26	2.29	2.26	2.30	2.26	2.28	2.20	2.21
other endocrine diseases	rest of E00–90	2.29	2.29	2.23	2.21	2.23	2.12	2.21	2.19	2.29	2.24	2.28	2.40	2.29
schizophrenia	F20–29	2.33	2.28	2.20	2.26	2.28	2.23	2.37	2.56	2.45	2.63	2.74	2.91	2.98
other psychiatric diseases	rest of F00–99	2.30	2.31	2.21	2.34	2.23	2.17	2.28	2.32	2.31	2.27	2.44	2.48	2.54
neurological diseases	G00–99	2.87	2.97	3.03	2.79	2.95	2.82	3.09	3.21	3.09	3.17	3.42	3.20	3.39
cataract	H25–26	2.13	2.15	2.03	2.05	2.04	2.05	2.02	1.97	1.99	2.07	2.12	1.95	1.97
other ophthalmic diseases	rest of H00–59	1.97	1.87	1.95	1.87	1.79	1.87	1.86	1.90	1.84	1.86	1.85	1.91	1.93
diseases of the ear	H60–95	2.50	2.65	2.50	2.64	2.53	2.44	2.34	2.49	2.45	2.54	2.51	2.47	2.47
hypertension	I10–15	2.14	2.10	2.09	2.05	2.08	2.08	2.11	2.14	2.19	2.25	2.32	2.35	2.36
ischemic heart diseases	I20–25	2.37	2.36	2.31	2.15	2.36	2.35	2.55	2.49	2.69	2.59	2.80	2.82	2.75
cerebrovascular diseases	I60–69	2.51	2.37	2.44	2.54	2.63	2.70	2.70	2.87	3.04	3.15	3.14	3.04	3.20
other circulatory diseases	rest of I00–99	2.60	2.61	2.53	2.67	2.57	2.59	2.69	2.64	2.79	2.78	2.78	2.91	2.88
acute upper respiratory infection	J00–06	2.01	2.03	2.06	2.06	2.02	2.07	2.04	1.94	1.99	1.96	1.94	2.04	2.00
pneumonia	J12–18	2.73	2.58	2.49	2.85	2.94	2.58	2.70	2.83	2.92	2.72	2.97	2.73	2.71
acute bronchitis	J20–21	2.04	2.07	2.14	2.00	1.94	1.94	2.01	1.95	1.92	1.89	1.96	2.07	2.09
chronic sinusitis	J32	2.48	2.38	2.47	2.30	2.55	2.39	2.51	2.30	2.33	2.45	2.45	2.41	2.48
chronic obstructive pulmonary diseases	J40–44	2.70	2.46	2.77	2.73	2.79	2.33	2.84	2.61	2.86	3.27	3.22	3.24	3.28
asthma	J45–46	2.51	2.57	2.52	2.52	2.50	2.50	2.55	2.60	2.56	2.54	2.71	2.67	2.71
other respiratory diseases	rest of J00–99	2.82	2.44	2.52	2.55	2.48	2.57	2.88	3.11	3.12	3.02	3.08	2.81	2.88
stomach and duodenal diseases	K25–29	2.36	2.41	2.33	2.39	2.42	2.39	2.45	2.40	2.45	2.46	2.59	2.59	2.65
liver diseases	K70–77	2.45	2.43	2.40	2.39	2.45	2.51	2.41	2.35	2.56	2.53	2.73	2.48	2.46
other gastrointestinal diseases	rest of K00–93	3.07	2.92	2.93	3.01	2.98	2.90	3.09	3.15	3.09	3.32	3.08	3.34	3.30
skin diseases	L00–99	2.71	2.54	2.48	2.32	2.32	2.31	2.39	2.32	2.25	2.33	2.39	2.33	2.43
musculoskeletal diseases	M00–99	2.53	2.51	2.48	2.49	2.52	2.55	2.53	2.51	2.42	2.47	2.45	2.50	2.50
genitourinary diseases	N00–99	3.46	3.52	3.56	3.59	3.92	3.89	3.86	3.79	3.84	4.55	4.50	3.87	4.55
pregnancy and delivery	O00–99	1.97	2.14	2.04	2.00	2.08	2.06	2.09	2.44	2.20	2.08	2.14	2.19	2.04
perinatal conditions	P00–96	2.81	3.72	2.72	1.90	2.29	2.40	1.97	3.04	1.78	2.40	2.90	2.43	2.35
congenital anomalies	Q00–99	2.63	3.54	2.73	3.24	4.23	3.27	3.10	2.67	3.70	2.68	3.20	2.24	3.60
unspecified conditions	R00–99	2.74	2.97	2.97	2.70	2.72	2.73	2.79	2.58	2.93	2.88	2.87	2.79	2.76
bone fractures	^a^	2.74	2.74	2.60	2.66	2.71	2.61	2.96	2.68	2.58	2.71	2.70	2.63	2.81
other injuries	rest of S00–T98	2.38	2.31	2.28	2.17	2.32	2.33	2.47	2.29	2.21	2.31	2.27	2.33	2.39

^a^S02,12,22,32,42,52,62,72,82,92,T02,08,10,12.

**Figure 1.**
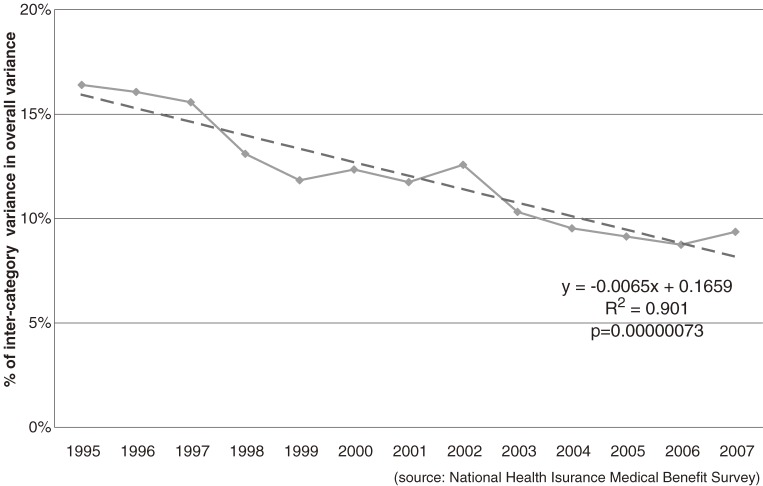
Trend in %intercategory variance in overall variance of per-claim cost of outpatient claims

Table 7 shows the consistent decline in interclass variance relative to overall variance: in 1995, intercategory variance was 19.5% of overall variance but declined to only 10% in 2007. This means that disease categories account for less than before in discriminating differences in per-claim costs; Figure 1 shoes the trend line (Y = −0.0065X + 0.1659, R^2^ = 0.901). The declining trend reversed in 2002, when hospitals and clinics were mandated to choose principal diagnoses; however, the reversal was only temporary and the declining trend appears consistent. In 2007, another reversal occurred, but it is too early to determine if it is temporary.

**Table 7.** Estimated variances of log-transformed per-claim costs

Year	Overall V (A)	Intercategory V (B)	Intracategory V	%intercategory V (B/A)
1995	53 023	8692	44 472	19.5%
1996	51 777	8315	44 595	18.6%
1997	52 011	8094	43 767	18.5%
1998	53 941	7064	47 376	14.9%
1999	57 383	6789	50 860	13.3%
2000	60 250	7440	53 551	13.9%
2001	64 965	7623	58 774	13.0%
2002	64 950	8162	59 730	13.7%
2003	70 608	7285	63 281	11.5%
2004	72 096	6873	67 171	10.2%
2005	76 907	7028	70 856	9.9%
2006	78 663	6876	71 910	9.6%
2007	81 517	7635	76 409	10.0%

V: variance.

## DISCUSSION

This study demonstrated a consistent decline in the intercategory variance of per-claim costs. If the difference in per-claim costs among disease categories is held constant, the declining intercategory variance can be interpreted as declining accuracy of classification or, in other words, increasing misclassification. Until 2001, disease classification was conducted rather arbitrarily, with no explicit criteria, by nonprofessionals at insurers. Starting in 2002, hospitals and clinics were required to specify principal diagnoses, which, it was hoped, would enhance the accuracy of classification. The change in classifiers did increase intercategory variance, as suggested by this author’s previous study,^17^ but the effect was short-lived and does not appear to have altered the overall declining trend. This finding is sufficient to rebut the common claim that classification is accurate when doctors choose principal diagnoses.

The goodness-of-fit evaluated by the KS test revealed that all disease categories followed log-normal distributions in 1995 and 1996, but that the goodness-of-fit deteriorated year by year as more categories were evaluated that did not follow a log-normal distribution, as indicated by increasing KS values. At the same time, this study revealed that the standard ratio of per-claim costs remained stable and close to the Napier constant (2.718). This finding should prove to be a useful rule of thumb for analysis of health insurance claims: 68% of claims fall between 2.718 times and 2.718th of the geometric mean.

**Figure 2.**
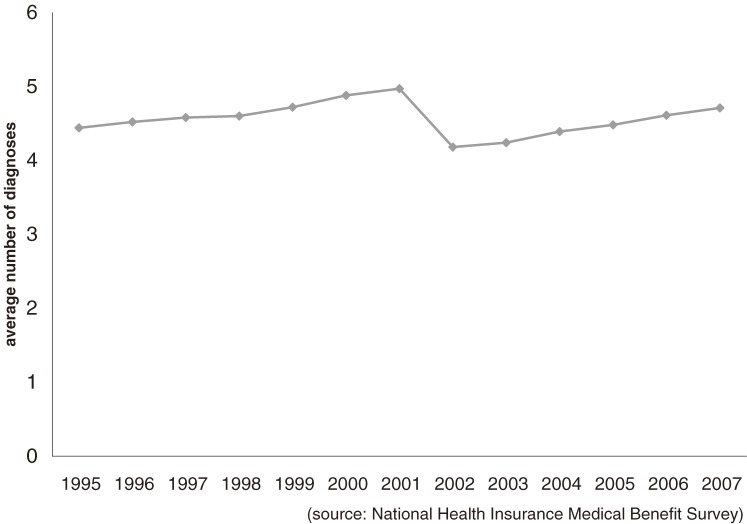
Trend in average number of diagnoses in an outpatient claim

Then, what is the cause of the decline in accuracy? The most probable cause is the increasing number of diagnoses, as suggested by this author in 1996.^13^ The average number of diagnoses in a claim has consistently increased, as shown in Figure 2. The increased intercategory variance in 2002 can be explained by the sudden reduction in the number of diagnoses due to the revised rule exempting diagnoses for inexpensive drugs. Whatever the causes, disease classification by principal diagnoses is becoming progressively less accurate in discriminating per-claim costs. With the rapid computerization of claims, there is a need for a statistical method that can objectively quantify all diagnoses. Such a method was described by this author in a previous study.^18^
